# Peer support working: a question of ontology and epistemology?

**DOI:** 10.1186/s13033-023-00570-1

**Published:** 2023-01-26

**Authors:** Michael John Norton

**Affiliations:** National Engagement and Recovery Lead, St. Loman’s Hospital, Palmerstown, Dublin, Republic of Ireland

**Keywords:** Epistemology, Lived experience, Ontology, Peer support working, Recovery

## Abstract

Mental health services are currently undergoing immense cultural, philosophical, and organisational change. One such mechanism involved in this change has been the recognition of lived experience as a knowledge subset in its own right. Within five Community Health Care Organisations [CHOs] in the Irish mental health services, 2017 marked a new era as the traditional statutory mental health service hired a total of 30 Peer Support Workers. Since then, additional Peer Support Workers were recruited along with the added addition of Family Peer Support Work. The purpose of such positions is to use their lived experiences and the knowledge subset within it to normalise experiences, break down hierarchical barriers and facilitate candid conversations that will allow the service user to progress on their own, self-defined recovery journey. Since it's inception into Irish mental health services, peer support has been line managed by a non-peer discipline. It is this where this paper highlights a potential problem. The paper raises concerns that the supervision conducted by these non-peer professionals could tamper, mutate and destroy the essence of peer support—the transfer and use of lived experience between service users. As such, a recommendation is suggested that the literature pauses discussions as to the mechanism by which lived experience is delivered and instead focus energies on identifying the ontological and epistemological position that underpins the experiences.One potential position to examine is that of constructionism as such knowledge is created or constructed through the fusion of life experiences and sub-concious thoughts and emotions experienced at a particular moment in time which are then entangled together with current information to create a narrative or story that can be therapeutic. It is through this philosophical exercise involviong/including existential themes that the essence of lived experience can be identified, protected, and nourished within mental health discourse.

## Introduction

The presence of the peer support profession within mental health service provision is not a new phenomenon. It dates as far back as psychiatry itself, which was formally identified as a medical speciality and discipline in the mid-1800s [1, 2]. However, in recent years it has exploded in popularity and is now observed as a mechanism used to create a recovery orientated service [3, 4]. In line with other jurisdictions, Ireland began to explore peer support as a treatment option and indeed profession in the early 2000s. Within an Irish context, the peer support movement began in County Mayo, Ireland, where several service users were offered training and employment in the role as part of a wider pilot project. However, despite these early advancements within a mental health service, the publication of vital guidance documents, like Naughton and colleagues [5] guidance paper on peer support working and the will of individuals involved in the Advancing Recovery in Ireland [ARI] initiative, it was not until February 20th 2017, that Peer Support Workers [PrSWs] were formally introduced into statutory mental health service provision [6]. Since then, the role has grown in popularity both academically and practically. With their ever-increasing presence, PrSWs have supported academics and services alike by closing the knowledge gap, caused by the over-reliance on what we call learned knowledge [7]. There are many examples of how this has occurred. On a multi-disciplinary team [MDT] level, PrSWs ensure that the service user’s voice is heard through supporting them to understand and actively contribute to their care plan. Additionally, PrSWs, through their presence alone, has been shown to cause other health care professionals to question and reflect on their own practice, behaviour and communication with service users [8]. Within the academic environment, PrSWs are starting to make their presence known in the education of other health care professionals, particularly those within the space of allied health care. Such impact is also noted within a number of evaluations such as Hunt and Byrne's [8] impact study and O'Dwyer O'Brien [9] doctoral research. Both of which found favourable results for PrSWs. As a direct response to the favourable outcomes experienced by both user and system, a toolkit was also co-produced to support the continuous implementation of the role [10]. Today, there are 29 active PrSWs and 4 Family Peer Support Workers [FPrSWs] in situ within the Irish traditional statutory mental health services. Additionally, there is an unknown number of both PrSWs and FPrSWs actively involved in other sectors relating to health, such as non-governmental organisations and sector 39 organisations like Bealach Nua and HAIL. However, despite such advances, peer support work still faces many organisational, structural, and philosophical challenges in today's mental health services.

## Personal experiences

I was lucky enough to become one of the first PrSW to be employed by the traditional mental health services in Ireland in 2017, when it was first rolled out. At that time, we began with 30 peers, all of which were eager to learn and to utilise their lived experiences to support the personal recovery journeys of many. During the first six to eight months of the post, we had a blended crash course in peer support working in mental health which involved academic work and practical time out in the community supporting individuals. Additionally, during this time, I was researching in the area of peer support for a Master's degree through research. This allowed me to intrinsically link my learning through research to inform my practice and thus for me my role was clear.

However, for many in this discipline, their views reflected that of the literature regarding role clarity, or in this case the lack of role clarity [11, 12]. Unfortunately, some five years later, issues of role clarity remain. The addition of FPrSWs to the services has also caused increase confusion and frustration between peers particularly around this issue of role clarity and the differences between the two peer roles. All of which is further hampered by PrSWs being supervised by other, non-peer professionals, particularly social work. Despite evidence to the contrary suggesting that this arrangement actually benefits the peer support profession [13]. In my opinion this has hampered the authenticity of peer support being delivered currently in an Irish setting. Here is why.

## Lived experience as a knowledge base

One of the central tenets of the peer role is the sharing of the lived experiences of the PrSW to the service user [8] A reason for this is the creation of informality that allows for three concepts to develop: the normalising of experiences, the development of a mutual, non-hierarchical safe space and the idealisation of the peer as a role model for recovery throught the identification of common experiences [14, 15]. These three concepts are imperative to the success of the peer intervention. However, this also requires us to formally recognise lived experience as a knowledge subset in its own right. In peer support, this knowledge subset is known as experiential knowledge. It is within this sphere that lies our problem. In order to truly understand and utilise lived experience/experiential knowledge within our services, the academic debate needs to stop examining how lived experience is being delivered [16, 17], and instead focus efforts first on the philosophical underpinnings of lived experience, for instance the ontological and epistemological positioning of lived experience in mental health discourse.

In essence, ontology refers to what is knowledge whereas epistemology is the study of how knowledge is created, validated and applied. Such discussions are vital as peers need to protect the essence of lived experience in their role. After all this is what exactly differentiates peers to their non peer counterparts. This paper argues that although peers and their non-peer counterparts are both working towards the same ideal, their ontological stance is different as the knowledge base used is different. However, they may exhibit the same epistemological position. Unlike peers, they develop their knowledge base through reading about conditions. However, where both groups are similar is in the application of such knowledge in practice as both must examine such knowledge, and intertwine it with the situation presented to them in a moment in time in order to therapeutically support the person—whether that be through the sharing of similarities [PrSWs] or through the sharing of clinical knowledge [non-peers]. As discussed in another paper, the results of not having this discussion and not providing protective measures for lived experience is the misinterpretation and under-utilisation of the role [15]. In terms of protective measures for the peer’s ontological and epistemological position, there firstly needs to be an acknowledgement of the differences in knowledge and an appreciation on both sides as to the importance of both knowledge sub-sets. Once this occurs, power needs to be equally shared between both parties. Although supervision is potentially a safe space for peers to be themselves, it is a relationship with a power hierarchy. This hierarchy needs to be neutralised or at least lessened in order for this knowledge set to be protected within a space where supervision occurs with non-peer staff. However, Irish services among others, need to also take ownership as to how peer support work was introduced into systems, particularly relating to their supervision and governance mechanisms. This is important as it can be argued within an Irish context, that five years of supervision from a profession that ultimately may have a different ontological and epistemological assumption to what constitutes knowledge has someway tampered with the potential such roles could have on our services.

## Conclusion

Although the concept of peer support is not new, the process underpinning it was not taken seriously or even valued until recently, when mental health services decided to create the role of PrSWs and FPrSWs. Peer support is based on the sharing of experiential knowledge attained through lived experience to break down hierarchical barriers and facilitate discussions on recovery. The importance of this process to the success of peer support interventions cannot be overstated. However, it is through service implementation of the role that a very real threat faces peer support. That is the transference of a different ontological and epistemological position onto PrSWs and FPrSWs which can negatively influence their practice. This paper suggests that the discussion within mental health discourse needs to change from how do we utilise lived experience to actually examining the philosophical basis of experiential knowledge so that the knowledge base created by same can be protected, harnessed and appropriately delivered within mental health service provision. One possible direction for future research is through the philosophical underpinnings of constructionism. Accorsing to Edwards and Titchen, as cited by Norton [18], constructionism is a view of the social world that is constructed from our various arrays of understanding. In this context, it is suggested that lived experience/experiential knowledge is constructed from one’s lived experiences of mental ill health, their sub-conscious thoughts and emotions experienced at a particular moement in time. Each of which are entangled with current knowledge of the service user’s situation so that a narrative can develop that can be therapeutic. However, more research and thought is required in order to truly understand if this position actually aligns with that of lived experience or not.

## Data Availability

Data sharing is not applicable to this article as no datasets were generated or analysed during the current study.
